# Association between neutrophil–lymphocyte ratio and arterial stiffness in patients with acute coronary syndrome

**DOI:** 10.1042/BSR20190015

**Published:** 2019-05-03

**Authors:** Yanyan Li, Xiaofang Chen, Lingzhi Huang, Jinyang Lu

**Affiliations:** 1 Department of Outpatient, Xiangya Hospital, Central South University, Changsha, Hunan Province 410008, China; 2 Department of Nursing, The Third Affiliated Hospital of Southern Medical University, Guangzhou, Guangdong Province 510630, China; 3 Clinical Nursing Teaching and Research Section, The Second Xiangya Hospital, Central South University, Changsha, Hunan Province 410011, China; 4 Department of Cardiovascular Medicine, The Second Xiangya Hospital, Central South University, Changsha, Hunan Province 410011, China; 5 Intervention Catheterization Room of Radiology Department, The Second Xiangya Hospital of Central South University, Changsha, Hunan Province 410011, China

**Keywords:** acute coronary syndrome, arterial stiffness, neutrophil-lymphocyte ratio, pulse wave velocity

## Abstract

The aim of the present study was to assess the association between neutrophil–lymphocyte ratio (NLR) and arterial stiffness and provide a predictive index for diagnosing atherosclerosis in patients with acute coronary syndrome (ACS). We enrolled patients with ACST who were confirmed by coronary angiography. Data were collected by questionnaire and blood indexes. Brachial-ankle pulse wave velocity (baPWV) was measured using BP-203RPE III network arteriosclerosis detection equipment. Correlation analysis of traditional cardiovascular risk factors and baPWV was performed, and multivariate line regression analysis was conducted to explore the relevant factors for baPWV. A total of 210 patients were included in the final analyses according to the inclusion criteria. Patients with a high baPWV had a lower lymphocyte count than those with a low baPWV (1.2 ± 0.4 vs. 1.4 ± 0.4, *P* = 0.004). The NLRs of the low and high bvPWV groups were 3.1 ± 1.5 and 4.0 ± 2.1, respectively; no significant difference was observed. The results suggest that there is a positive relationship between baPWV and NLR (*r* = 0.403, *P* = 0.005) and neutrophils (*r* = 0.319, *P* = 0.016). Multivariate line regression suggested that NLR was positively associated with baPWV (*B* = 0.372, *P* = 0.000). The present results indicate that NLR is independently associated with arterial stiffness in patients with ACS. NLR, an inexpensive, easily measurable, widely available biomarker, could be an additional tool for assessing cardiovascular risk in clinical practice.

## Introduction

The burden of atherosclerotic cardiovascular disease (ASCVD) has increased rapidly and substantially in China. There were approximately 2.4 million deaths from ASCVD in 2016, representing 61% of deaths from cardiovascular disease (CVD) and 25% of all deaths, which is an increase from ∼1 million deaths from ASCVD (40% of deaths from CVD and 11% of all deaths) [1]. Acute coronary syndrome (ACS) is a common and severe cardiovascular and coronary heart disease (CHD) [2]. The release of inflammatory cytokines and the inflammatory response are critical pathophysiological factors [3]. Occasionally, ACS can be caused by non-atherosclerotic diseases such as arteritis, trauma, arterial dissection, thromboembolism, congenital variation, cocaine abuse, or cardiac catheterization complications. These patients usually have an abrupt onset, and the degree of risk varies. The keys to improving prognosis are early diagnosis, timely risk stratification, and reasonable clinical intervention [4].

Pulse wave velocity (PWV) is a noninvasive and simple method for accurately assessing the structure and function of arteries and vessel walls [5]. A rapid PWV shows that the degree of arterial stiffness is strong and that the compliance of blood vessel walls is poor. A slow PWV means that the arterial vessel has a low stiffness and the vessel wall has good compliance [6,7]. Early atherosclerosis can be observed by detecting PWV. Arterial stiffness increases with decreased compliance, which can be observed before obvious symptoms appear. This method can provide a favorable opportunity for clinical treatment [8,9]. The neutrophil–lymphocyte ratio (NLR) is a biomarker that reflects the inflammation status of the human body [10] as well as the balance between activation and regulatory factors of inflammation [11]. Several studies have found that NLR is associated with malignant tumors [12], coronary artery disease [13], atherosclerosis [14], heart failure [15], acute pancreatitis [16], diabetes [17], obesity and dyslipidemia [18], hypertension [19], metabolic syndrome (MetS) and endothelial dysfunction [20], osteoporosis [21], and polycystic kidney disease [22,23]. Researchers have increasingly realized the importance of NLR in the progression of ACS. A previous study suggested that neutrophils could infiltrate at the early stage into brittle plaques such as fibrous cap, shoulder patch, middle layer of vessel, endothelial, and capillary plaques with hemorrhage. Kalay et al. found that circulating neutrophil count and NLR were associated with the severity of CHD [24]. Nilsson reported an elevated neutrophil count in lipid plaque cores from patients with CHD using coronary CT imaging [25]. However, few studies have assessed the relationship between NLR and baPWV in patients with ACS. The aim of the present study was to assess the association between NLR and arterial stiffness and provide a predictive index for diagnosing atherosclerosis in patients with ACS.

## Materials and methods

### Study population

We retrospectively collected the data of patients with ACS who were confirmed by coronary angiography from September 2016 to August 2017. The diagnosis of CHD was made according to the diagnostic criteria recommended by the International Heart Disease association and the diagnostic criteria for ACS recommended by the World Health Organization (WHO), American College of Cardiology/American Heart Association (ACC/AHA), and European Society of Cardiology ESC ACSs [26,27]. ACS can describe chest pain felt during a heart attack, while at rest, or when doing light physical activity. The ST segment changes and persists for more than 20 min after taking nitroglycerin. Coronary angiography shows that coronary stenosis was >50% (unstable angina) in at least one primary coronary artery. The criteria for acute myocardium infarction were as follows: troponin was typically elevated and gradually decreased with at least one of the following items: (1) typical symptoms of myocardial ischemia (clinically, chest pain), severe dyspnea, and shock; (2) electrocardiogram showing pathological Q wave or ST segment elevation/downward movement; and (3) coronary angiography or imaging showing vascular stenosis. The exclusion criteria were diabetes, hypertension, stroke, artery thrombosis, phlebitis, tumor, acute and chronic infection, trauma, and severe liver and kidney dysfunction.

The study was approved by the Ethics Committee of The Third Affiliated Hospital of Southern Medical University (2018060032). The participants of the study were informed of the study aim and that the whole process would not have privacy disclosure. The research was carried out in accordance with the World Medical Association Declaration of Helsinki, and all subjects provided written informed consent.

### Demographic data

Demographic data were obtained using a standard questionnaire. The following information was collected: age, sex, body mass index (BMI), history of smoking, and history of diabetes. Systolic blood pressure (SBP) and diastolic blood pressure (DBP) were measured, and mean arterial pressure (MAP) was calculated using the following formula: MAP = (SBP + 2DBP)/3.

### Biochemical measurements

Biochemical parameters were measured using an automated Beckman Coulter LH-750 Hematology Analyzer (Beckman Coulter, Inc., Fullerton, CA, U.S.A.). Hemoglobin, microalbuminuria, total cholesterol, total triglyceride, high-density lipoprotein cholesterol (HDL-C), low-density lipoprotein cholesterol (LDL-C), blood urea nitrogen (BUN), uric acid, estimated glomerular filtration rate (eGFR), high-sensitivity C-reactive protein (hsCRP), white blood cell (WBC), red blood cell, neutrophils, lymphocytes, and NLR were measured. Patient general characteristics were determined using a standard questionnaire administered by a group of trained investigators. Urine microalbuminuria concentrations were measured using the immunoturbidimetric method. BMI was calculated as weight (kg) divided by height squared (m^2^). eGFR was calculated using the formula: 186 × SCr^–1.154^ × age in years^–0.203^ × 1.210 (if black) × 0.742 (if female) [28]. MAP was calculated as one-third SBP and two-thirds DBP. Two experienced physicians conducted coronary arteriography to detect coronary stenosis, and quantitative assessments followed the Gensini accumulated method.

### Examination of arterial stiffness

Brachial-ankle pulse wave velocity (baPWV) was used to evaluate arterial stiffness after hospital admission. Patients rested for 15 min without smoking in a quiet room at an appropriate temperature before testing. Then, baPWV was measured using an Omron waveform analyzer (BP-203RPE III) by placing oscillometric sensors at the ends of the arms and ankle. Each study subject was tested twice, and the mean of the two results was recorded. The normal reference range for baPWV is <1400 cm/s, and a baPWV > 1400 cm/s indicates increased arterial stiffness [29].

### Statistical analysis

The study population was divided into high and low baPWV groups. Continuous variables were expressed as means and standard deviations if the data had a normal distribution, and *t* tests were used. For continuous variables with a non-normal distribution, non-parametric tests were used. Classification data were expressed as percentages and counts, and two groups were compared using chi-square tests. Pearson’s correlation coefficient was used to assess the relationship between baPWV and quantitative parameters, and Spearman’s correlation coefficient was used to assess baPWV and qualitative data. A partial correlation coefficient was also calculated between baPWV and NLR with adjusted potential factors. Multivariate line regression analysis was conducted to explore the relevant factors for baPWV. baPWV was treated as the dependent variable and age, sex, BMI, smoking, diabetes, MAP, hemoglobin, microalbuminuria, total cholesterol, total triglyceride, eGFR, hsCRP, WBC, red blood cell, neutrophils, lymphocytes, and NLR were independent variables. All statistical analyses were performed using SPSS 22.0 software version, and *P* < 0.05 was considered statistically significant.

## Results

A total of 210 patients were included in the final analyses according to the inclusion criteria; 120 and 90 patients in the low and high baPWV groups, respectively. The age of the study population ranged from 36 to 65, with a mean of 57.1 years. The mean ages of the low and high baPWV groups were 52.3 ± 14.6 and 63.6 ± 11.8, respectively. The percentage of female patients in the low baPWV group was 50%, compared with 60% in the high baPWV group. The NLR of the low group was 3.1 ± 1.5, compared with 4.0 ± 2.1 in the high bvPWV group; this difference was statistically significant. Compared with the low baPWV group, patients in the high baPWV group tended to be older (63.6 ± 11.8 vs. 52.3 ± 14.6, *P* < 0.001), have a higher BMI (22.1 ± 3.4 vs. 20.8 ± 4.1, *P* = 0.015), have more diabetes (30.0% vs. 10.0%, *P* = 0.002), and elevated SBP and MAP levels (*P* = 0.026 and *P* = 0.019, respectively). Total cholesterol and uric acid levels were higher (*P* = 0.000 and *P* = 0.002, respectively) and the lymphocyte count was lower (*P* = 0.004) in the control group. No significance differences were found in other parameters (Table 1).

**Table 1:** General characteristics between two groups

Factors	Low baPWV (*n* = 120)	High baPWV (*n* = 90)	*t/χ*^2^/*u*	*P*
Age (year)	52.3 ± 14.6	63.6 ± 11.8	6.014	<0.001
Sex (female%)	60 (50.0)	54 (60.0)	2.072	0.150
BMI (kg/m^2^)	20.8 ± 4.1	22.1 ± 3.4	2.443	0.015
Smoking (%)	30 (25.0)	24 (26.7)	0.074	0.784
Diabetes (%)	12 (10.0)	27 (30.0)	13.603	0.002
Hypertension (%)	48 (40.0)	40 (44.4)	0.417	0.518
SBP (mmHg)	128.3 ± 17.5	132.5 ± 15.4	−2.242	0.026
DBP (mmHg)	71.3 ± 16.8	75.1 ± 12.6	−1.799	0.073
MAP (mmHg)	90.3 ± 17.8	94.2 ± 1593	−1.681	0.094
Heart rate (*n*)	67.3 ± 7.9	69.1 ± 8.6	−1.572	0.117
ACS type			0.157	0.924
STEMI	31 (25.8)	23 (25.6)		
NSTEMI	30 (25.0)	20 (22.2)		
Other	59 (49.1)	37 (41.1)		
Hemoglobin (g/L)	104.3 ± 24.6	99.7 ± 18.9	1.477	0.141
Microalbuminuria (g/L)	32.8 ± 5.0	31.9 ± 5.6	0.149	0.798
Total cholesterol (mmol/L)	5.3 ± 1.2	4.7 ± 1.0	3.846	0.000
Total triglyceride (mmol/L)	1.7 ± 0.9	1.6 ± 1.0	−0.620	0.536
HDL-C (mmol/L)	1.57 ± 0.4	1.53 ± 0.4	0.035	0.972
LDL-C (mmol/L)	3.6 ± 0.8	3.74 ± 0.8	−1.222	0.902
Uric acid (mmol/L)	312.6 ± 49.6	333.4 ± 46.9	−3.007	0.002
BUN (mmol/L)	4.8 ± 1.1	4.8 ± 1.2	0.000	0.999
eGFR (ml/min/1.73 m^2^)	5.8 ± 3.1	6.2 ± 2.7	−0.977	0.330
hsCRP (mg/dl)	1.2 (0.8–4.1)	4.7 (0.7–10.6)	2.367	0.345
WBC (×10^9^/mm^3^)	6.4 ± 1.5	6.3 ± 2.2	0.391	0.696
Red blood cell (×10^12^/L)	5.0 ± 40.4	5.1 ± 36.8	−0.018	0.985
Neutrophils (×10^3^/mm^3^)	4.3 ± 1.2	4.2 ± 1.6	0.518	0.605
Lymphocyte (×10^3^/mm^3^)	1.4 ± 0.4	1.2 ± 0.4	−2.928	0.004
NLR	3.1 ± 1.5	4.0 ± 2.1	3.622	0.000
baPWV (cm/s)	1646.4 ± 220.6	2476.2 ± 588.2	14.189	<0.001

STEMI: St-segment elevation myocardial infarction; NSTEMI: non-St-segment elevation myocardial infarction.

The correlations between baPWV and other parameters in patients with ACS are presented in Table 2. There was an obvious positive relationship between baPWV and NLR (*r* = 0.403, *P* = 0.005) and neutrophils (*r* = 0.319, *P* = 0.016). There was a negative correlation between baPWV and lymphocyte count (*r* = –0.206, *P* = 0.030). Age, smoking, diabetes, SBP, MAP, hs-CRP, LDL-C, uric acid, and WBC were positively correlated with baPWV (Table 2). The partial correlation coefficient between baPWV and NLR via adjusting potential factors was 0.398 (*P* = 0.013).

**Table 2:** Correlation analysis between factors and baPWV

Factors	*r*	*P*
Age (year)	0.786	<0.001
BMI (kg/m^2^)	−0.193	0.434
Smoking (%)	0.361*	0.002
Diabetes (%)	0.431*	0.004
Hypertension (%)	0.138	0.612
SBP (mmHg)	0.512	0.021
DBP (mmHg)	0.108	0.681
Heart rate (*n*)	0.115	0.593
MAP (mmHg)	0.354	0.015
Hemoglobin (g/L)	−0.076	0.521
Microalbuminuria (g/L)	−0.421	0.006
Total cholesterol (mmol/L)	−0.186	0.196
Total triglyceride (mmol/L)	−0.095	0.403
HDL-C (mmol/L)	−0.123	0.361
LDL-C (mmol/L)	0.201	0.042
Uric acid (mmol/L)	0.623	0.000
BUN (mmol/L)	0.034	0.726
rGFR (ml/min/1.73 m^2^)	0.168	0.072
hsCRP (mg/dl)	0.305	0.007
WBC (×10^9^/mm^3^)	0.291	0.035
Red blood cell (×10^12^/L)	0.107	0.367
Neutrophils (×10^3^/mm^3^)	0.319	0.016
Lymphocyte (×10^3^/mm^3^)	−0.206	0.030
NLR	0.403	0.005

* Spearman correlation coefficient.

Multivariate line regression analysis was performed by treating baPWV as the dependent variable and age, sex, BMI, smoking, diabetes, MAP, hemoglobin, microalbuminuria, total cholesterol, total triglyceride, eGFR, hsCRP, WBC, red blood cell, neutrophils, lymphocytes, and NLR as independent variables. The results suggested that NLR was positively associated with baPWV (B = 0.372, *P* = 0.000). baPWV increased by 0.372 when NLR changed by a unit. In addition, age, SBP, BMI, total cholesterol, and uric acid were related to baPWV (*B* = 0.517, *P* = 0.000; *B* = 0.462, *P* = 0.001; *B* = 0.499, *P* = 003; *B* = 2.530, *P* = 0.022; and *B* = 3.381, *P* = 0.000, respectively) (Table 3).

**Table 3:** Multivariate line regression of relevant affecting baPWV*

Factors	*B*	SE	*t*	*P*
Age	0.517	0.496	6.387	0.000
SBP	0.462	0.130	3.445	0.001
NLR	0.372	3.120	7.384	0.000
BMI	0.499	0.176	2.982	0.003
Total cholesterol	2.530	1.098	2.304	0.022
Uric acid	3.381	0.481	8.071	0.000

* Adjusted *R*^2^ = 0.432.

## Discussion

The present study found that patients in the high baPWV group had an increased NLR compared with those in the low baPWV group. Multivariate line regression analysis suggested that NLR was an independent predictor of baPWV in patients with ACS.

Atherosclerosis is the pathological basis of macrovascular diseases, and neutrophils are a key factor in the inflammatory response. On the one hand, neutrophils can strengthen inflammatory reactions by increasing macrophage and antigen levels. On the other hand, neutrophils participate in acute tissue damage by secreting inflammatory mediators [30]. For example, neutrophils release arachidonic acid derivatives and superoxide radicals by proteolytic enzymes, which make the formed plaques more fragile. Activated neutrophils also adhere to the endothelial cell surface and cause endothelial cell dysfunction. During this process, the up-regulation of pro-inflammatory factors further aggravates vascular inflammation, and long-term inflammation leads to vascular smooth muscle proliferation, microvascular formation, and subsequent arteriosclerosis [31]. Previous studies found that platelet-induced inflammatory responses played a key role in the formation of atherosclerosis. Activated platelets can release inflammatory mediators and mitotic substances into the local microenvironment [31], which recruit more platelets and WBCs to the inflammatory sites such as platelet C3a and C5a-induced inflammatory factor-17. This process could accelerate arteriosclerosis [32]. Because the total number of plasma lymphocytes in patients with ACS decreased and the CD4/CD8 ratio decreased significantly, the decline in the number of lymphocytes reduced the body’s anti-inflammatory ability, creating a short-term inflammatory status [33]. Therefore, some studies reported that lymphopenia could be associated with the progress of atherosclerosis

NLR reflects the ratio of neutrophils to lymphocytes. The current results suggest that baPWV was significantly associated with BMI and total cholesterol, despite no significant correlations between baPWV with BMI and total cholesterol. There was no significant difference in neutrophils between the high and low baPWV groups, whereas the lymphocyte count was decreased significantly in the high baPWV group compared with the low baPWV group. It is possible that the increase in NLR was caused by the decreased lymphocyte count, which is consistent with a report by Solak et al. [34]. NLR can also reflect the stability of the WBC count, and cannot be affected by changes in acute state. Patients with ACS usually have varying inflammatory statuses, and so blood cells can affect atherosclerosis in different ways [35]. A previous study found that in the inflammatory state, neutrophils are an important regulatory factor in the progression of tissue damage to the arterial wall. Lymphocyte apoptosis further increased artery hardening [36]. In addition, low lymphocyte levels may be associated with malnutrition, and neutrophil and lymphocyte counts were significantly associated with PWV in patients with hematologic malignancies [37]. The comprehensive analysis above shows that reduced lymphocyte levels and elevated neutrophil levels both predict the severity of arteriosclerosis in different ways.

The main difference between the present study and the previous report is that the present study was conducted in patients with ACS. The present study has several limitations. First, it was conducted in patients with ACS, and so caution must be used when applied to other population settings. Second, the present study had a cross-sectional design, and thus a cause–effect relationship cannot be determined. Third, the present study did not define the pathology mechanism. Finally, some parameters such as creatine kinase, creatine kinase-MB, and cardiac troponin I were not included in the analysis, and these parameters may affect the results. Therefore, further studies are needed.

In conclusion, the present results demonstrated that NLR was independently associated with arterial stiffness in patients with ACS. NLR, an inexpensive, easily measurable, widely available biomarker, could be an additional tool for assessing cardiovascular risk in clinical practice. However, further prospective studies are needed to confirm a cause-and-effect relationship.

## Abbreviations

ACS: acute coronary syndrome
ASCVD: atherosclerotic cardiovascular disease
baPWV: brachial-ankle pulse wave velocity
BMI: body mass index
BUN: blood urea nitrogen
CHD: coronary heart disease
DBP: diastolic blood pressure
GFR: glomerular filtration rate
HDL-C: high-density lipoprotein cholesterol
hsCRP: high-sensitivity C-reactive protein
LDL-C: low-density lipoprotein cholesterol
MAP: mean arterial pressure
NLR: neutrophil–lymphocyte ratio
SBP: systolic blood pressure
WBC: white blood cell
